# Exact Solution to an Interacting Extreme-Value Problem: The Pure-Flaw Model

**DOI:** 10.6028/jres.099.031

**Published:** 1994

**Authors:** P. L. Leath, P. M. Duxbury

**Affiliations:** Department of Physics and Astronomy, Rutgers University, Piscataway, NJ 08855-0849; Department of Physics and Astronomy, Michigan State University, East Lansing, MI 48824-1116

**Keywords:** extreme-value distributions, scaling analysis, size effect in fracture

## Abstract

Simple models play a key role in the microstructural analysis of mechanical failure in composites and other materials with complex and often disordered microstructures. Although equal load-sharing-models are amenable to rigorous statistical analysis, problems with local load enhancements near failed regions of the material have so far resisted exact analysis. Here we show for the first time, that some of the simpler of these *local-load*-*sharing models* can be solved exactly using a sub-stochastic matrix method. For diluted fiber bundles with local load sharing, it is possible to find a compact expression for the characteristic equation of the sub-stochastic matrix, and from it obtain an asymptotic expansion for the largest eigenvalue of the matrix. This in turn gives the asymptotic behavior of the size effect and statistics of the fiber-bundle models. We summarize these results, and show that the important features of the exact result can be obtained from a single scaling analysis we had developed previously. We also compare the statistics of fracture with the appropriate limiting extreme-value survival distribution (a Gumbel distribution), and, as previously indicated by Harlow and Phoenix, note that the Gumbel distribution performs quite poorly in this problem. We comment on the physical origin of this discrepancy.

## 1. Introduction

It has been known since the pioneering work of Chaplin [1] and well known since the classic work of Griffith [2] that randomly occurring flaws or weak links effectively determine the observed tensile strength of materials. Early on it was realized that the dependence of failure upon the weakest part of a material structure gives rise to non-Gaussian statistics for fracture stress and strain. These developments lead to the classical period of the development of the statistics of extremes by mathematicians such as Dodd [3], Frechet [4], Fisher and Tippett [5], von Mises [6], Gnedenko [7], and Gumbel [8].

Following the work of Duxbury et al. [9–11], there have been many attempts to use random network models to determine the statistics and size dependence of material breakdown [12–16]. These calculations have in many cases elucidated the general behavior and size dependence of breakdown, but few exact results have been produced.

**Fig. 1:**
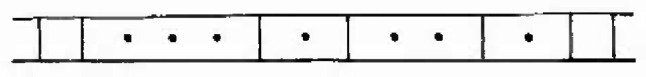
A one dimensional array of intact bonds (fibers) and flaws (vacancies). The tensile stress *σ*, is applied vertically.

Perhaps the simplest model that shows the statistics of brittle failure has been the pure-flaw, chain-of-bundles model of Harlow and Phoenix [17] which has been studied by Harlow [18] and more recently by Harlow and Phoenix [19]. In this model there is a series or chain of *m* structurally and statistically independent bundles of *n* elements each as shown in Fig. 1, where the vertically applied uniaxial stress is shared by the surviving vertical fibers (bonds). Each element or fiber is independently present with probability *p* and absent with probability *f*=1−*p*. The survival probability of the chain of bundles is then the survival probability of a single bundle raised to the power *m*. The main difficulty in this analysis is calculating the survival probability of a single bundle. The extension of this theory to the survival of two-dimensional networks is straightforward and amounts to the approximation that cracks or flaws only exist and break along the direction transverse to the direction of the applied stress. Following Harlow and Phoenix, we assume the local-load-sharing model for a flaw (crack) of length *n* to be

$$\sigma_{\mathrm{tip}}=\sigma_{0}\left(1+n/2\right),$$ (1)

which is to say that the entire force applied to the cluster is concentrated at the tip (on the fibers adjacent to each end of the flaw or vacancy cluster). Failure of any surviving bond (all of which have the same strength) leads to a rip which causes failure of the entire bundle. Solution of this model requires finding the bond (weakest link) which experiences the largest stress enhancement and that stress which would break this most stressed bond.

**Fig. 2:**
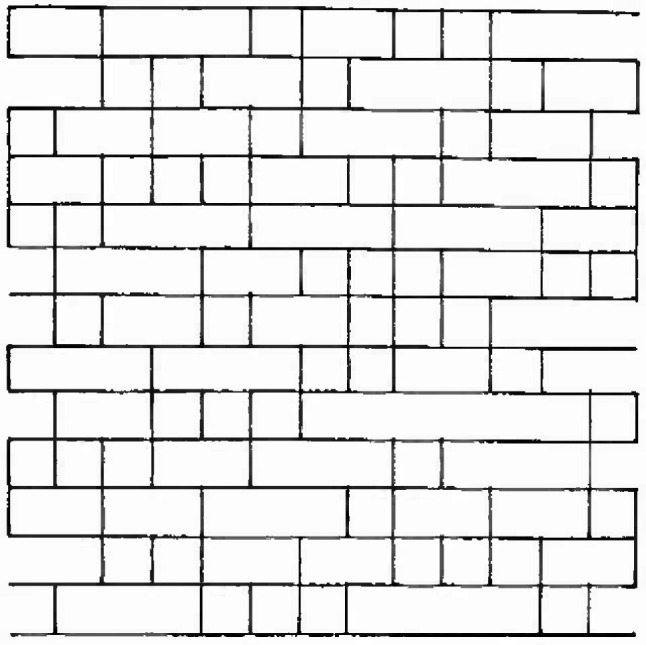
A two dimensional lattice with only horizontal cracks. Spiral boundary conditions identify each site on the right edge with the site on the left edge of the previous row.

Duxbury, Leath, and Beale [11] showed how a one-dimensional model such as this could be used as a simple model for fracture or breakdown of a two-dimensional network. If one considers that cracks or flaws only exist and break horizontally, then the two-dimensional model becomes that illustrated in Fig. 2 (i.e., no horizontal bonds break). Then if we impose spiral boundary conditions, where the last site in a row is connected to the first site of the previous row on the other side of the sample, then the *N* × *N* network problem is reduced to a one-dimensional chain of *N*^2^ fibers (or bonds) in parallel like that in Fig. 1.

## 2. Single-Cluster Calculation: The Sub-Stochastic Transition-Matrix Method

In this calculation one assumes that the weakest link is the pure flaw or cluster of vacancies of the largest size that exists in the sample. The survival probability is then closely related to the probability that in a sample of length *L*(= *N*^2^) that there are *no* clusters of vacant bonds (flaws) of size greater than some prescribed value *n*. Using a generating-function technique Duxbury, Leath, and Beale [11] calculated exactly the asymptotic form of the probability to be

$$C_{L}\left(n\right)\approx {\left[1-pf^{n+1}\right]}^{l}.$$ (2)

in the limit of large *L*. It is now possible to rederive this result while introducing the sub-stochastic transition-matrix method. Following Harlow [18] we define all possible endings of a fiber bundle of length *L* + 1 and the way in which those endings may be generated from a bundle of length *L* and the probabilities of those endings. Since there are no allowed clusters larger than *n*, the allowed bundle endings or distinctive endings at a particular site are an occupied site (1) followed by 0≤*r*≤*n* vacancies (0) so that these distinct endings are spanned by the basis vector $\phi_{L}^{T}={\left(\phi_{\left(1\right)},\phi_{\left(10\right)},\phi_{\left(100\right)}\ldots .,\phi_{\left(10\ldots 0\right)}\right)}_{L}$ where the last element contains *n* zeros.

Then the probability increment for going from a cluster of size *r* to a cluster of size *r′* on the next site is included in the matrix product

$$M\phi_{j}=\left[\begin{array}{ccccc} p & p & p & \ldots & p\\ f & 0 & 0 & \ldots & 0\\ 0 & f & \ddots & \ddots & \vdots \\ \vdots & \vdots & \ddots & 0 & 0\\ 0 & 0 & \ldots & f & 0 \end{array}\right]\;{\left[\begin{array}{c} \phi_{\left(1\right)}\\ \phi_{\left(10\right)}\\ \vdots \\ \vdots \\ \phi_{\left(10\ldots 0\right)} \end{array}\right]}_{j}={\left[\begin{array}{c} \phi_{\left(1\right)}\\ \phi_{\left(10\right)}\\ \vdots \\ \vdots \\ \phi_{\left(10\ldots 0\right)} \end{array}\right]}_{j+1},$$ (3a)

which is the same as the matrix *M* operating *j* times on the probability vector *ϕ*_0_ for the starting site, or

$$M\phi_{j}=M^{j+1}\;\phi_{0}.$$ (3b)

The probability *C_L_*(*n*) that there are only clusters up to size *n* in the entire bundle (or network) of size *L* is thus

$$C_{L}\left(n\right)=\sum_{\ell }{\left(\phi_{\ell }\right)}_{L},$$ (4)

where the sum is over all the elements of *ϕ_L_*. The simplest and most natural boundary conditions are periodic ones where *C_L_*(*n*) becomes the trace

$$C_{L}\left(n\right)=tr\left(M^{L}\right)=\sum_{k}\lambda_{k}^{L},$$ (5)

since the 1st and *L* th sites must be the same, where *λ_k_* are the eigenvalues of *M*. We find the eigenvalues of *M* via its characteristic equation

$$D_{n}=\det|M/\lambda -I|=\left|\begin{array}{ccccc} \left(a-1\right) & a & \cdots & a & a\\ b & -1 & 0 & \ldots & 0\\ 0 & b & \ddots & \ddots & \vdots \\ \vdots & \ddots & \ddots & -1 & 0\\ 0 & \ldots & 0 & b & -1 \end{array}\right|=0,$$ (6)

where *a* = *p/λ* and *b* = *f/λ* = (1 − *p*)/*λ*. A cofactor expansion of the determinant *D_n_* about its last row, gives immediately the recursion relation

$$D_{n}=-D_{n-1}+{\left(-1\right)}^{n}\;ab^{n},$$ (7a)

where *D_n_*_−1_ is the *n* × *n* determinant for clusters up to size (*n* − 1). With *D*_0_ = *(a* − 1), the solution, upon iteration of Eq. (7a), is the characteristic equation

$${\left(-1\right)}^{n}D_{n}⇌\left(a-1\right)+ab+ab^{2}+\cdots +ab^{n}=0.$$ (7b)

Summing this geometric series we obtain

$$\lambda^{n+2}-\lambda^{n+1}+pf^{n+1}=0.$$ (8)

This equation is the characteristic equation of *M* times (*λ* − *f*), so there is an additional spurious root at *f* (since we are interested in the largest root, this does not affect the analysis). Since *M* is primitive and non-negative, its largest eigenvalue is real and distinct and it can easily be seen that all the eigenvalues are less than 1 and λ_max_ approaches 1 for large *n*, Thus we set λ_max_ = 1 − ϵ and expand Eq. (8) to lowest order in ϵ and *f^n^*, which gives us

$$\lambda_{\max}\approx 1-pf^{n+1}+O\left(f^{2n}\right).$$ (9)

Then, we obtain

$$C_{L}\left(n\right)≅\lambda_{\max}^{L}≅{\left(1-pf^{n+1}\right)}^{L},$$ (10)

which confirms the result Eq. (2) by the sub-stochastic transition-matix method.

In order to find the failure probability as a function of applied stress, we use the load-sharing rule Eq. (1), coupled with the fact that the failure of the bond carrying the largest local stress nucleates catastrophic failure, and thereby use the relation

$$\sigma_{b}/\sigma =1+\frac{n}{2},$$ (11)

where *σ*_b_ is the breaking strength of a single fiber. Note that we could have used a variety of other load-sharing rules here, and for example the same expression with *n* raised to an arbitrary power is also of physical interest. This result combined with Eq. (10) yields the probability *S* (*σ*) that a fiber bundle will survive at stress *σ*

$$S\left(\sigma \right)={\left(1-pf^{2\frac{\sigma_{b}}{\sigma }-1}\right)}^{L}.$$ (12)

for large *n* and *L*, this becomes the modified Gumbel form, introduced previously [10, 11] in the analysis of the random fuse network. Although *C_L_*.(*n*) in Eq. (10) becomes a Gumbel distribution in n, the substitution of *n* (*σ*) from Eq. (11) produces a modified Gumbel distribution that is significantly different from a Gumbel form in *σ* in the high-reliability tail of the distribution. This modification is discussed further in Sec. 4.

## 3. Double-Cluster Calculation

**Fig. 3:**
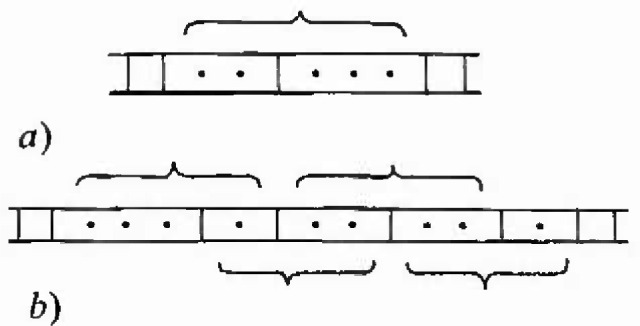
**a**) A double cluster of *≤ n* total vacancies plus one isolated occupied bond, **b**) Repeated, overlapping double dusters; each pair of clusters as indicated by the brackets contains ≤ *n* total vacancies plus one isolated bond.

Several authors [12, 16, 19, 20] have suggested that the most critical defect is not a single cluster of *n* vacancies, but rather a double cluster (double co-linear crack) of *n* vacancies separated by a single occupied site located anywhere within the *n* + 1 adjacent sites. Such a double crack is shown in Fig. 3a. This candidate for the most critical crack is appealing because the stress enhancement at the interior occupied site grows as *n* in network models rather than as *n*^1/2^ (as for the edges of a single crack in a two-dimensional network) and because the increased entropy of the (*n* + 1) locations of the occupied site makes it more probable. Thus, following Harlow and Phoenix [19], we consider the probability of bundles of length *L* with repeated double cracks (and single cracks when the occupied site is at either end) not exceeding *n* vacant sites in any two adjacent cracks separated by a single site. These repeated double cracks are shown in Fig. 3b.

Harlow [18] showed that this problem is amenable to analysis by the sub-stochastic transition-matrix method. The load-sharing rule is still given by Eq. (1) as before but now *n* is the sum of the number of vacant sites immediately on the left and right of any isolated intact bond or fiber. Thus it is necessary to keep track of not only the number of vacancies in the cluster being considered but also those in the previous vacant cluster. There are now *(n* + l)(*n* +2)/2 distinct endings that must be considered at a site (or bundle ending); these are given by the basis vector *ϕ* = *ϕ*_(1)_, *ϕ*_(10)_…*ϕ*_(10…0)_; *ϕ*_(101)_, *ϕ*_(1010)_…*ϕ*_(1010…0)_;*ϕ*_(1001)_…*ϕ*_(10010…0)_;…; *ϕ*_(10…01)_ where there are no more than *n* total vacancies in any element. With this ordering of states the *n* = 4 sub-stochastic matrix for this problem, for example, is given by Eq. (13).

With periodic boundary conditions Eq. (5) still holds and we again analyse the largest eigenvalue of *M_n_*. As a technical point, note that since we are using periodic boundary conditions, we can always start the matrix process at a surviving bond, and so the endings considered above include all possible

$$M_{4}\phi_{L}=\left[\begin{array}{ccccccccccccccc} p & 0 & 0 & 0 & 0 & p & 0 & 0 & 0 & p & 0 & 0 & p & 0 & p\\ f & 0 & 0 & 0 & 0 & 0 & 0 & 0 & 0 & 0 & 0 & 0 & 0 & 0 & 0\\ 0 & f & 0 & 0 & 0 & 0 & 0 & 0 & 0 & 0 & 0 & 0 & 0 & 0 & 0\\ 0 & 0 & f & 0 & 0 & 0 & 0 & 0 & 0 & 0 & 0 & 0 & 0 & 0 & 0\\ 0 & 0 & 0 & f & 0 & 0 & 0 & 0 & 0 & 0 & 0 & 0 & 0 & 0 & 0\\ 0 & p & 0 & 0 & 0 & 0 & p & 0 & 0 & 0 & p & 0 & 0 & p & 0\\ 0 & 0 & 0 & 0 & 0 & f & 0 & 0 & 0 & 0 & 0 & 0 & 0 & 0 & 0\\ 0 & 0 & 0 & 0 & 0 & 0 & f & 0 & 0 & 0 & 0 & 0 & 0 & 0 & 0\\ 0 & 0 & p & 0 & 0 & 0 & 0 & f & 0 & 0 & 0 & 0 & 0 & 0 & 0\\ 0 & 0 & 0 & 0 & 0 & 0 & 0 & 0 & 0 & 0 & 0 & p & 0 & 0 & 0\\ 0 & 0 & 0 & 0 & 0 & 0 & 0 & 0 & 0 & f & 0 & 0 & 0 & 0 & 0\\ 0 & 0 & 0 & 0 & 0 & 0 & 0 & 0 & 0 & 0 & f & 0 & 0 & 0 & 0\\ 0 & 0 & 0 & p & 0 & 0 & 0 & 0 & p & 0 & 0 & 0 & 0 & 0 & 0\\ 0 & 0 & 0 & 0 & 0 & 0 & 0 & 0 & 0 & 0 & 0 & 0 & f & 0 & 0\\ 0 & 0 & 0 & 0 & p & 0 & 0 & 0 & 0 & 0 & 0 & 0 & 0 & 0 & 0 \end{array}\right]\;\left[\begin{array}{l} \phi_{\left(1\right)}\\ \phi_{\left(10\right)}\\ \phi_{\left(100\right)}\\ \phi_{\left(1000\right)}\\ \phi_{\left(10000\right)}\\ \phi_{\left(101\right)}\\ \phi_{\left(1010\right)}\\ \phi_{\left(10100\right)}\\ \phi_{\left(101000\right)}\\ \phi_{\left(1001\right)}\\ \phi_{\left(10010\right)}\\ \phi_{\left(100100\right)}\\ \phi_{\left(10001\right)}\\ \phi_{\left(100010\right)}\\ \phi_{\left(100001\right)} \end{array}\right]=\phi_{L+1}$$ (13)

survival configurations (we don’t have to consider configurations which start with 0’s).

A great simplification in the characteristic equation

$$M_{n}\phi =\lambda \phi ,$$ (14)

where *ϕ* are the eigenvectors of *M_n_*, is possible since most of the rows of *M_n_* contain only a single non-zero element *f*. This gives, for example *fϕ*_(1)_ = λ*ϕ*_(10)_ or *bϕ*_(1)_ = (*f*/λ)*ϕ*_(1)_ = *ϕ*_(10)_ By such relations, we can eliminate all the rows of *M_n_* except the rows with *p’*s corresponding to the reduced basis vector *ϕ* = *ϕ*_(1)_, *ϕ*_(101)_, *ϕ*_(1001)_,…, *ϕ*_(100…01)_. The resulting (*n*+1) equations give an (*n* +l) × (*n* + 1) matrix *M*′, which satisfies the reduced characteristics equation

$$M\prime \phi =\left[\begin{array}{cccccc} a & a & a & \ldots & \ldots & a\\ ab & ab & \ldots & \ldots & ab & 0\\ ab^{2} & ab^{2} & \ldots & ab^{2} & 0 & \vdots \\ ab^{3} & ab^{3} & ⋰ & 0 & \ldots & \vdots \\ \vdots & ⋰ & ⋰ & \ldots & \ldots & 0\\ ab^{n} & 0 & 0 & \ldots & 0 & 0 \end{array}\right]\;\left[\begin{array}{c} \phi_{1}\\ \phi_{2}\\ \phi_{3}\\ \vdots \\ \vdots \\ \phi_{n+1} \end{array}\right]=\left[\begin{array}{c} \phi_{1}\\ \phi_{2}\\ \phi_{3}\\ \vdots \\ \vdots \\ \phi_{n+1} \end{array}\right],$$ (15)

where *a* =*p*/*λ*, and *b* = *f*/λ. This *M’* matrix can be considered as a new transition-matrix which adds a *cluster* at a time rather than a bond or fiber at a time. Thus we have the characteristic determinant equation.

$$D_{n}=\det\left|\begin{array}{cccccc} a-1 & a & a & \ldots & \ldots & a\\ ab & ab-1 & \ldots & \ldots & ab & 0\\ ab^{2} & ab^{2} & ab^{2}-1 & ⋰ & ⋰ & \vdots \\ ab^{3} & ab^{3} & ⋰ & \ddots & & \vdots \\ \vdots & ⋰ & ⋰ & \ldots & -1 & 0\\ ab^{n} & 0 & 0 & \ldots & 0 & -1 \end{array}\right|=0.$$ (16)

For small *n* these determinants can be evaluated directly. For example,

$$\begin{array}{l} D_{0}=-1+a,D_{1}=1-a-a^{2}b\;\text{and}\\ D_{2}=-1+a+ab+a^{2}b^{2}-a^{3}b^{3}. \end{array}$$ (17)

But by expanding the determinants Eq. (16) by rows and columns, we can show that there is an exact recursion relation

$$bD_{n}\left(a,b\right)=sD_{n-2}\left(ab,b\right)-D_{n-4}\left(ab^{2},b\right),$$ (18)

where *D_m_*(*ab^ℓ^,b*) is *D_m_*(*a*,*b*) with *a* replaced by (*ab^ℓ^*), where

$$s=1+b-a^{2}b^{n+1}.$$ (19)

Note that *s* =*s*(*a,b,n*) = *s*(*ab*,*b*,*n*−2) = *s*(*ab*^2^,*b*,*n*−4) which is key in the solvability of the recursion relation Eq. (18). After some detailed analysis, we have found (see [21] for details), that this recurrence equation may be solved. The resulting characteristic equation is given by

$$\frac{{z_{+}}^{\left(n-1\right)/2}-{z_{-}}^{\left(n-1\right)/2}}{{z_{+}}^{\left(n+1\right)/2}-{z_{-}}^{\left(n+1\right)/2}}=s-\left(1+ab^{\left(n+1\right)/2}\right),$$ (20a)

for *n* ⩾3 and odd. While for *n* even ⩾4, we find,

$$\frac{{z_{+}}^{n/2-1}-{z_{-}}^{n/2-1}}{{z_{+}}^{n/2}-{z_{-}}^{n/2}}=s-\frac{1}{1-ab^{n/2}}.$$ (20b)

In Eqs. (20a) and (20b),

$$Z_{+,-}=\frac{s\pm \sqrt{s^{2}-4b}}{2b}.$$ (20c)

The key quantity *s* is given by Eq. (19) above. Equations (20a,b) are the exact expressions for the characteristic equation of the original *M* in Eqs. (13) and (14).

Again we find that the largest eigenvalue of *M* is near 1 for large *n*, so we set λ = 1 − *ϵ* and expand Eqs. (20a, b) and find that in both cases, to lowest order in *ϵ* and *f^n^*,

$$\epsilon =\left[\left(n+2\right)p^{2}-p\right]f^{n+1}+O\left(f^{3n/2}\right).$$ (21)

Comparing this double-cluster result to the single-cluster result Eq. (9) we find the expected (*n* +2) from the possible locations of the single bond in a double cluster of size (*n* + 1). The (−*p*) is a correction to properly handle the single-cluster cases as well as the double-cluster case, since these are included whenever the isolated bond is located at one end of the double cluster. It is only important for small *n*.

**Fig. 4:**
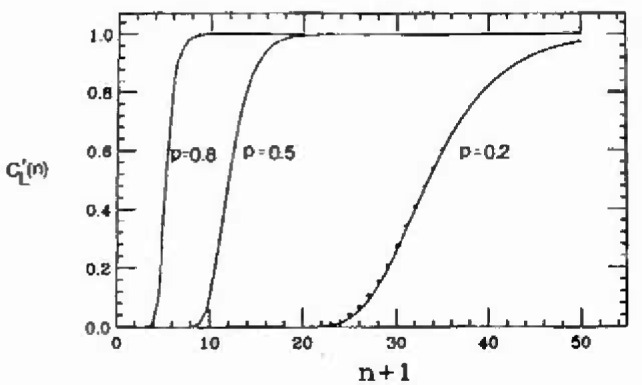
A plot of the double-cluster probability $C\prime_{L}\left(n\right)\;\text{vs}\;\left(n+1\right)$, for *p* − 0.2, 0.5, and 0.8. The dotted line is the asymptotic form given by Eq. (24). The solid lines are found from evaluating the largest eigenvalue numerically, and by using $C\prime_{L}\left(n\right)=\lambda_{\max}^{L}$.

**Fig. 5:**
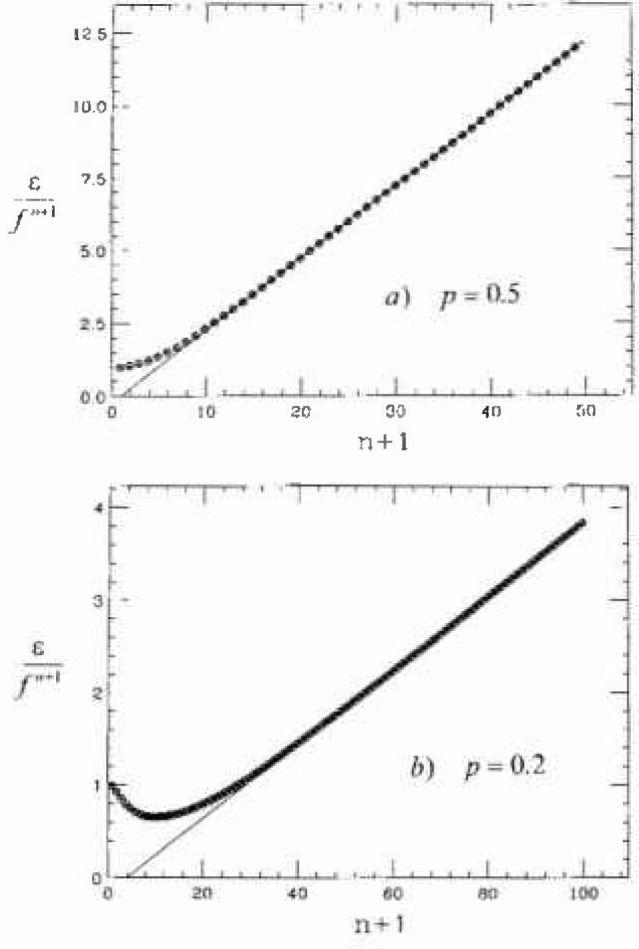
Plots of ϵ/*f^n^*^+1^ vs *n* + 1, for a) *p* = 0.5 and b) *p* = 0.2. The solid lines are the asymptotic form as given by Eq. (22); the circles arc the exact values of $\left(1-\lambda_{\max}^{L}\right)/f^{n+1}$ as obtained by iterating *M* numerically.

In order to check and better understand the asymptotic form Eq. (21) of λ_max_ and the importance of the other eigenvalues λ*_i_* we have made several numerical evaluations of the various equations. First, we have numerically found the largest eigenvalue λ_max_ of the original, full, sub-stochastic transition-matrix *M* as given by Eq. (13). Using the unit vector as a starting vector we repeatedly apply the matrix *M* to it. Since the largest eigenvalue is unique, this process converges exponentially to the largest eigenvalue. We found in general that convergence occurred to six significant figures with at most 50 matrix products (even for matrices *M* of dimension (*n* + 1)(*n* +2)/2 = 10,000. The sparsity of *M* with this iterative procedure eliminated matrix-storage problems. The results of this iterative procedure for $C\prime_{L}\left(n\right)$ versus *n* are shown in Fig. 4 as solid lines, and the dots give $\lambda_{\max}^{L}$ with λ_max_ as given by the asymptotic form Eq. (21). Good agreement is seen for all *p*, with a small deviation in the *p* =0.2 data. However, a more stringent test is needed in the high-reliability (large *n*) tail. Thus, in Fig. 5 we plot the quantity

$$\frac{1-\lambda_{\max}}{f^{n+1}}=\frac{\epsilon }{f^{n+1}}\approx \left[\left(n+2\right)p^{2}-p\right].$$ (22)

The solid straight line versus (*n* + 1) is the asymptotic result, which is linear in *n* and this is compared with the iterated numerical values (circular symbols), for *p* = 0.5 and 0.2. For large *n*, in all cases the two calculations agree. But for small values of *p*<0.5 there appears a minimum in (1 − λ_max_)/*f^n^*^+1^ versus *n* which corresponds to higher order terms in *f^n^*. In particular the next order term in $\lambda_{\max}^{L}$ is *O*(*f*^3^*^n^*^/2^) which would appear as a *O*(*f^n/^*^2^) correction in Fig. 5.

**Fig. 6:**
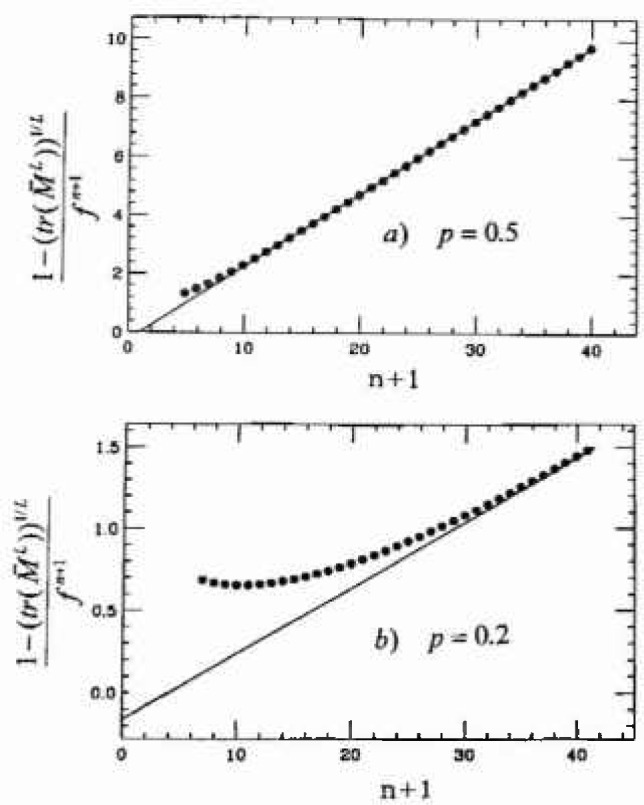
Plots of the exact value of [1−(*tr*(*M^L^*))*^t/L^*]/*f^n^*^+1^ vs (n+1) obtained numerically from *M* (circles); for *L* = 1000, for a) *p* = 0.5 and b) *p* =0.2. The solid line is the asymptotic form Eq. (22).

Finally, we test for the accuracy of neglecting all but the maximum eigenvalue $\lambda_{\max}^{L}$. It is possible to directly, numerically evaluate the trace of *M^L^* by iteration since it only requires storage of the matrix and one vector at any time. Using this method, we have evaluated the quantity

$$\left(1-{\left(tr\left(M^{L}\right)\right)}^{1/L}\right)/f^{n+1},$$ (23)

which should converge to (1 − λ_max_)/*f^n^*^+1^ when the largest eigenvalue is dominant. A numerical test of this convergence is shown in Fig. 6, for *L* = 1000, and shows that for large lattice sizes, the most important corrections to $C\prime_{L}\left(n\right)$ are the higher order contributions to λ_max_, which are of *O*(*f*^3^*^n/^*^2^), rather than the neglected smaller eigenvalues of *M*, which are relatively unimportant here.

Finally, we obtain the asymptotic form

$$C\prime_{L}\left(n\right)={\left\{1-\left[\left(n+2\right)p^{2}-p\right]f^{n+1}+O\left(f^{3n/2}\right)\right\}}^{L}.$$ (24)

Thus, following the same arguments as at the end of Sec. 2, we find that to leading order the survival probability of the entire network or chain-of-bun-dles is

$$S\prime_{L}\left(\sigma \right)={\left[1-\left(\frac{2\sigma_{b}}{\sigma }p^{2}-p\right)f^{\frac{2\sigma_{b}}{\sigma }-1}\right]}^{L},$$ (25)

which is again of the form of a modified Gumbel distribution with slightly different coefficients from Eq. (12).

## 4. Extreme-Statistical Form

For large *L*, we can easily find the limiting form of $C\prime_{L}\left(n\right)$ as given by Eqs. (10) and (24) respectively,

$$C_{L}\left(n\right)\underset{L\to \propto }{\sim }\exp\left[-Lp\;\exp\left(-n\;\log\left(1/f\right)\right)\right],$$ (26)

and

$$C\prime_{L}\left(n\right)\underset{L\to \propto }{\sim }\exp\left[-L\left(\left(n+2\right)p^{2}-p\right)\exp\left(-n\;\log\left(1/f\right)\right)\right],$$ (27)

as the upper limit behavior for large *n* which is important in the high-surviveability tail of the distribution. This is a Gumbel distribution, as is expected from the exponential [*pf^n^* or *nf^n^*] behavior for the probability of large clusters [Castillo, 1988].

On the other hand, the survivabilities *S*(*σ*), and *S*′(*σ*), in the limit *L*→∞, are found to be *modified* Gumbel forms [from Eqs. (12) and (25)],

$$S_{L}\left(\sigma \right)\underset{L\to \infty }{\sim }\exp\left[-Lp\;\exp\left(-\left(\frac{2\sigma_{b}}{\sigma }-1\right)\log\left(1/f\right)\right)\right]$$ (28a)

and,

$$\begin{array}{c} S\prime_{L}\left(\sigma \right)\underset{L\to \infty }{\sim }\exp[-L\left(\frac{2\sigma_{b}}{\sigma }p^{2}-p\right)\exp(-\left(\frac{2\sigma_{b}}{\sigma }-1\right)\\ \log\left(1/f\right))]. \end{array}$$ (28b)

That the dominant behavior of *S_L_*(*σ*), and $S\prime_{L}\left(\sigma \right)$ is $\exp\left(-LA\;\exp\left(-\frac{\partial }{\sigma }\right)\right)$ as *σ* tends to zero is essential to ensuring that the survival distributions have the proper limiting approach to one when the applied stress approaches zero. Harlow and Phoenix [19] have numerically shown that this high-reliability tail can not be well described by a Gumbel form for *σ* (such as exp(−*LA* exp(*B*(*σ* − *σ*_b_)))). But this failure is obvious since the Gumbel form doesn’t approach one until *σ →* − ∞, so at sufficiently small stresses it must be inaccurate. Nevertheless the standard texts on extreme distributions (see, for example [8] or [22]) seem to suggest that the Gumbel distribution is the appropriate one in such cases. The difference clearly is in the form of the normally assumed scaling lim*_N→α_*[*S*(*σ*)]*^N^*=*S*(*a_N_σ* + *b_N_*), which fits the shift and slope of the limiting function *S*(*σ*) at its median, but fails near the high-reliability limit *σ* = 0. It would seem that instead a more general scaling form lim*_N_*_→α_[*S*(*n*(*σ*))]*^N^* = *S*(*a_N_n*(*σ*) + *b_N_*) must be allowed to also include the proper high-surviveability limit near zero stress. In many practical material-failure problems, this modification of the Gumbel form is essential in order to correctly represent the important high-reliability tail. Note that this is not true of the Weibull distribution, which as well as being a stable limiting extreme-value distribution, does have the physically correct behavior as stress approaches zero. This is one good reason why the Weibull distribution is a very robust form in the analysis of failure problems. We suggest that the family of modified Gumbel distributions of the sort Eq. (28), should be similarly robust, in contrast to the conventional Gumbel distribution which is of limited use in the analysis of the statistics of material failure.

## 5. General Scaling Behavior

The size dependence and general form of the limiting distribution can usually be found from a back-of-the-envelope scaling calculation which we introduced previously [9, 11]. First, for the single-cluster calculation, consider the probability *P_L_*(*n*) of finding a cluster of size *n* in a sample of size *L*. The order of magnitude of this probability is

$$P_{L}\left(n\right)\propto Lp^{2}f^{n}/p=Lpf^{n},$$ (32)

since, for normalization, ∑*p*^2^*f*″=*p*, and since there are *L* different locations in the sample where a cluster could be located. For the maximum cluster size to be expected in a sample of size *L* we set

$$P_{L}\left(n\right)\sim Lpf^{n_{\max}}=1,$$ (33)

and obtain the size dependence

$$n\text{Max}≅\frac{\log L}{\log\left({\;}^{t}{/}_{f}\right)},$$ (34)

or, from the load-sharing rule Eq. (1), the breakdown stress *σ*_c_

$$\sigma_{c}^{\left(L\right)}\sim \frac{1}{1+n_{\max}/2}∼\frac{1}{1+\frac{\ln L}{2\ln\left(1/f\right)}},$$ (35)

scales to zero logarithmically in the thermodynamic limit. A similar argument for double clusters gives

$$P\prime_{L}\left(n\right)\propto L\left(n+1\right)p^{2}f^{n},$$ (36)

since there are (*n* + 1) places to put an isolated bond in the *n* -double-clusters. We then obtain the same limiting form Eq. (35) for *σ*_c_(*L*), although there are additive (log(log*L*)) corrections in the double-cluster case. The logarithmic scaling law in turn implies that the failure statistics is of the double-exponential form given in Eq. (28). The Weibull and Frechet distributions always give power-law size scaling. These qualitative arguments are very powerful and are confirmed by the rather elaborate, exact calculation described here.
